# Steady-State Delivery and Chemical Modification of Food Nutrients to Improve Cancer Intervention Ability

**DOI:** 10.3390/foods13091363

**Published:** 2024-04-28

**Authors:** Sijia Hao, Peng Ge, Wentao Su, Yuxiao Wang, A. M. Abd El-Aty, Mingqian Tan

**Affiliations:** 1 State Key Laboratory of Marine Food Processing and Safety Control, Dalian Polytechnic University, Dalian 116034, China; haosijia@bhu.edu.cn (S.H.); gepeng@dlpu.edu.cn (P.G.); suwt@dlpu.edu.cn (W.S.); wangyuxiao@lnu.edu.cn (Y.W.); 2 Academy of Food Interdisciplinary Science, School of Food Science and Technology, Dalian Polytechnic University, Dalian 116034, China; 3 National Engineering Research Center of Seafood, Dalian Polytechnic University, Dalian 116034, China; 4 Dalian Key Laboratory for Precision Nutrition, Dalian Polytechnic University, Dalian 116034, China; 5 Department of Pharmacology, Faculty of Veterinary Medicine, Cairo University, Giza 12211, Egypt; abdelaty44@hotmail.com; 6 Department of Medical Pharmacology, Medical Faculty, Ataturk University, Erzurum 25240, Turkey

**Keywords:** diet, nutritional intervention, steady state delivery, chemical modification, cancer

## Abstract

Cancer is a crucial global health problem, and prevention is an important strategy to reduce the burden of the disease. Daily diet is the key modifiable risk factor for cancer, and an increasing body of evidence suggests that specific nutrients in foods may have a preventive effect against cancer. This review summarizes the current evidence on the role of nutrients from foods in cancer intervention. It discusses the potential mechanisms of action of various dietary components, including phytochemicals, vitamins, minerals, and fiber. The findings of epidemiological and clinical studies on their association with cancer risk are highlighted. The foods are rich in bioactive compounds such as carotenoids, flavonoids, and *ω*-3 fatty acids, which have been proven to have anticancer properties. The effects of steady-state delivery and chemical modification of these food’s bioactive components on anticancer and intervention are summarized. Future research should focus on identifying the specific bioactive compounds in foods responsible for their intervention effects and exploring the potential synergistic effects of combining different nutrients in foods. Dietary interventions that incorporate multiple nutrients and whole foods may hold promise for reducing the risk of cancer and improving overall health.

## 1. Introduction

**Figure 1:**
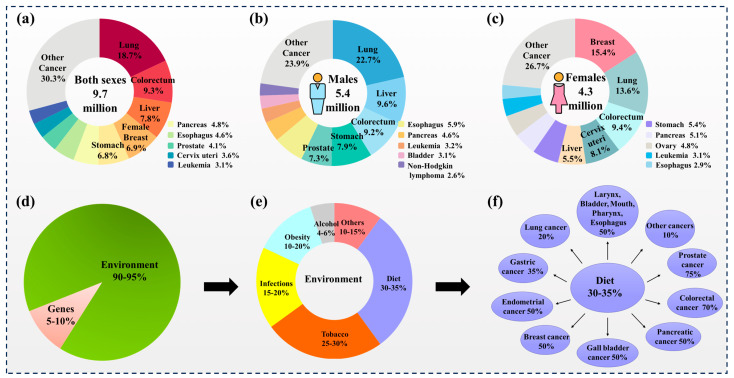
Distribution of cancer cases [1] and influencing factors [2]. Deaths for the most common cancers in 2020 for (**a**) Both sexes. (**b**) Males. (**c**) Females [1]. (**d**) The contribution of genes and the environment to the development of cancer. (**e**) The percentage contribution of every environmental factor. (**f**) The percentage of cancer deaths linked to diet [2].

Cancer is currently the second leading cause of death worldwide, after cardiovascular diseases such as ischemic heart disease. Based on the International Agency for Research on Cancer (IARC), there were probably 20.0 million new cases of cancer and 9.7 million deaths from cancer worldwide in 2022 [1]. Lung cancer is the most common cause of cancer death in the world, representing 18.7% of the total cancer mortality rate, followed by colorectal (9.3%), liver (7.8%), female breast (6.9%), and stomach (6.8%) cancers (Figure 1a–c) [1]. While genetic defects can play a role in developing some types of cancer, they are estimated to cause only 5–10% of all cancer cases (Figure 1d) [2]. Most cancers are believed to be caused by a combination of environmental and lifestyle factors, such as exposure to tobacco smoke, air pollution, UV radiation, an unhealthy diet, lack of physical activity, and infectious agents such as viruses and bacteria [2,3]. Epidemiological studies have suggested that changes in nutritional factors and dietary patterns could potentially prevent up to 35% of cancer cases. However, the actual percentage may vary depending on the specific dietary composition and the type of cancer [2] (Figure 1e,f). While cancer prevention and intervention strategies continue to evolve, current evidence suggests that adopting healthier habits can be important in reducing the risk of developing cancer and improving outcomes for those with cancer. These habits include avoiding excessive exposure to ultraviolet radiation and other carcinogens, such as tobacco smoke and environmental pollutants, reducing alcohol intake, maintaining a healthy diet, and engaging in regular physical activity [4,5,6].

**Figure 2:**
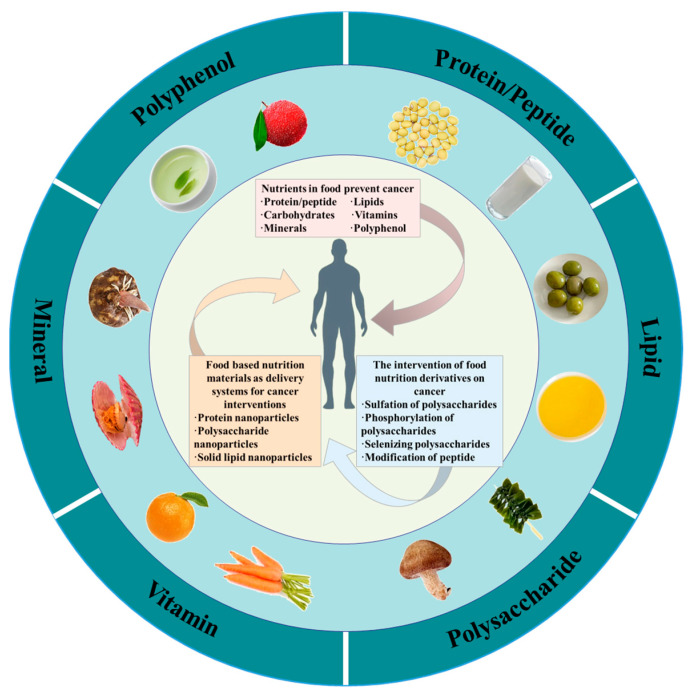
Summary of essential nutrients and their state delivery and chemical modification in cancer intervention.

Carcinogenesis is a multistep process that involves the transformation of the normal cell into the tumorigenic neoplastic cell. The pathogenic mechanisms involved in tumor initiation, promotion, and progression are diverse and complex [7]. There has been significant interest in the role of foods and nutrition in developing and managing cancer since the publication of studies in the early 1980s [8]. Over four decades, numerous observational research studies have investigated the relationship between nutrient intake and cancer risk. In general, these analyses have found that consumers who eat a diet rich in fruits, vegetables, whole grains, and other nutrient-dense foods are at lower risk of developing certain types of cancer compared to those who consume a diet high in processed foods, red and processed meats, and unhealthy fats [9,10,11]. Human cancer development is a complicated process that can take years or even decades. It is recommended that healthy diet and nutrition interventions for cancer prevention and management should start early in life and be sustained over time. Furthermore, nutritional supplementation can significantly impact the response to cancer treatment and the overall outcomes of cancer patients. Nutritional supplementation can help balance energy expenditure and nutrition intake, which is vital for maintaining body weight and preventing malnutrition during cancer treatment [12,13,14]. Nutritional supplementation can be an essential component of systematic therapy in cancer patients, as it can help to improve clinical symptom management and quality of life (Figure 2) [15,16].

**Figure 3:**
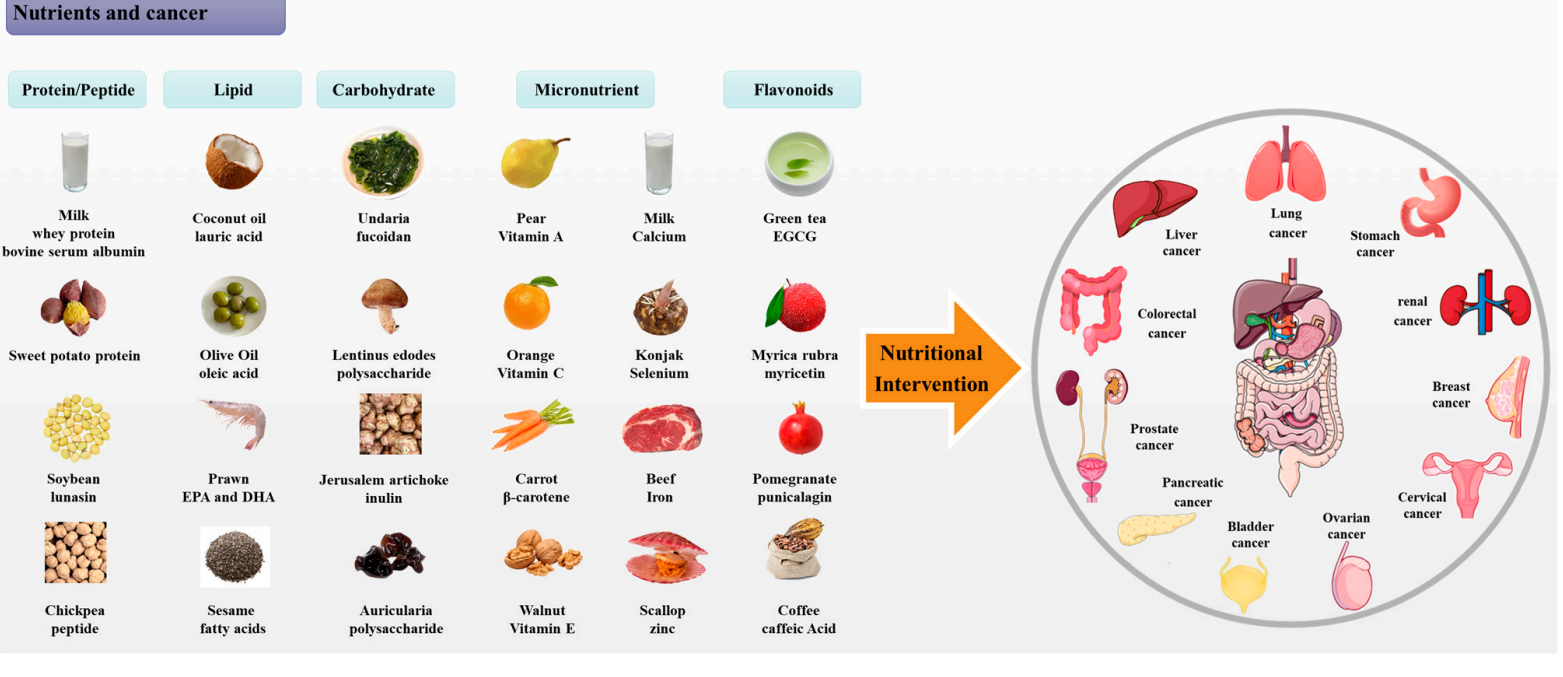
Summary of protein/peptides, lipids, carbohydrates, micronutrients, and flavonoids from foods with anti-cancer properties.

The focus of nutrient- and food-driven chemoprevention has been on identifying specific types of foods and nutrients that can potentially reduce the risk of cancer. This includes foods rich in protein, vitamins, minerals, and fiber. A healthy diet can contribute to the maintenance of a standard body weight, the enhancement of general health, and the reduction of the rate of chronic diseases such as cancer (Figure 3).

## 2. Nutrients in Food for Cancer Prevention

### 2.1. Protein/Peptides

**Figure 4:**
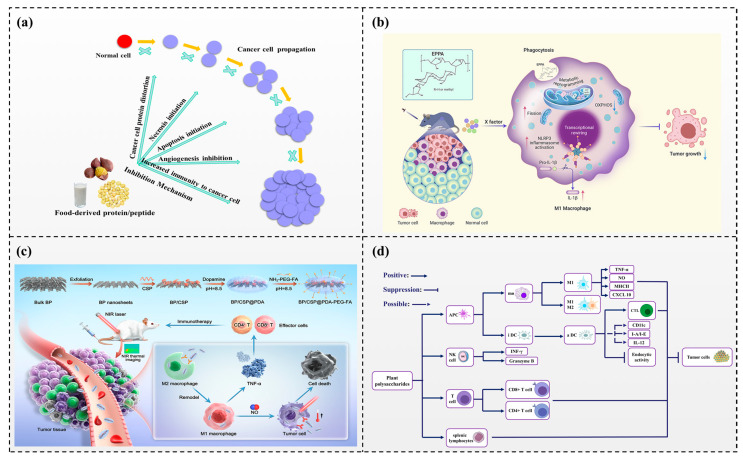
Nutrients in food and their potential in the intervention of cancer (**a**) Food-derived protein/peptides and their mechanism of action against cancer. (**b**) The systemic immune function of orally administered inulin gel [42] (reproduced with permission from publisher Elsevier). (**c**) The anti-tumor efficacy of echinacea purpurea-derived homogeneous polysaccharide [43] (reproduced with permission from publisher American Chemical Society) (**d**) Action pathways of plant polysaccharides [48].

Protein is a macronutrient that is important for the body to function properly. It is a source of essential amino acids and can be used as an energy source when carbohydrates and fats are unavailable. In addition to essential nutrition, some food-derived proteins contain bioactive peptides, short chains of amino acids that can have health benefits beyond their nutritional value [17]. The well-accepted mechanisms that can be used to fight cancer include inducing programmed cell death (apoptosis), antiangiogenesis, antimetastasis, function blocking, and immunomodulation. As the saying goes, “prevention is better than cure”. Food-derived antiproliferative proteins/peptides have shown promising potential in preventing cancer development. By inhibiting the growth of cancer cells, they can act as adjuvant chemoprevention compounds and reduce the risk of cancer [18] (Figure 4a).

Whey protein is a protein found in milk and is a byproduct of cheese-making. It is often used as a dietary supplement in oncology to help manage malnutrition and improve muscle mass in cancer patients undergoing treatment [19]. Cytokines are a diverse group of small proteins produced by various types of cells, including immune cells, and act as signaling molecules to regulate the immune response. Several cytokines play a part in regulating the inflammatory tumor microenvironment. Proinflammatory cytokines have been deemed to take effect in breast cancer [20]. The research report may be the basis for proposing innovative therapeutic approaches for breast cancer. Pupae protein, obtained from *Bombyx mori* or *Samia ricini*, might have anticancer potential by downregulating the expression of proinflammatory cytokines such as IL-6, IL-1β, and TNF-α [21]. Metastasis is a significant factor in cancer progression and is often associated with a poorer prognosis. Over the years, a wide range of natural and synthetic compounds have been identified as potential anti-metastatic agents that can help to prevent cancer cells from invading surrounding tissues and spreading to other parts of the body. In this context, Li et al. [22]. carried out research to investigate the potential anticancer effects of sweet potato protein against colorectal cancer cells. The researchers found that sweet potato protein was able to inhibit the growth and multiplication of human colonic cancer cells (SW480) in vitro and reduce the size of tumors in mice implanted with SW480 cells in vivo. The antiproliferative effects of sweet potato protein were mediated, at least in part, by the induction of apoptosis of malignant cells and inhibition of the uPA signaling pathway. These research results also provided the basis for the antitumor potential of food-derived protein extracts.

Food-derived bioactive peptides are typically produced through enzymatic hydrolysis of their parent protein, gastrointestinal digestion, and microbial fermentation [23]. Peptides are generally considered to have several advantages over larger proteins, including their low molecular weight, high affinity, strong specificity for the target, low toxicity, and good tissue penetration [24]. Peptides generally exhibit better bioavailability than their parent proteins due to their smaller size and increased solubility.

Existing research has investigated the mechanisms of anticancer peptides, which include a range of effects on tumor cells and their microenvironment, such as distortion of crucial proteins related to the proliferation of tumor cells, intervention of enzymatic activities related to tumor growth, immunity enhancement against tumor cells, suppression of the angiogenesis process of tumor cells, and initiation of necrosis or apoptosis. Bioactive peptides from food sources have been systematically proven to be suitable substitutes for tumor management [25]. Additionally, peptides from different food sources have demonstrated potential promising effects against tumors [26]. Lunasin was initially discovered in soybeans and other seeds and is a polypeptide consisting of 43 amino acids. Hsieh et al. [27] assessed the antitumor effects of lunasin on 7,12-dimethylbenz(a) anthracene and 3-methylcholanthrene-treated fibroblast cells (NIH/3T3). Lunasin significantly suppressed cell proliferation and reduced cancerous foci formation in these cells treated with two chemical carcinogens. Hsu et al. [28] found that the peptides LPHVLTPEAGAT and PTAEGGVYMVT, extracted by enzymatic hydrolysis of tuna dark muscle, had the effect of inhibiting the proliferation of MCF-7 breast cancer cells in vitro. This study suggested that these peptides could be used as natural anticancer agents or functional food ingredients. Furthermore, the decapeptide RQSHFANAQP has been proven to have strong antiproliferative properties on human breast cancer cells (MCF-7 and MDA-MB-231). The peptide was extracted from chickpea protein through enzymatic hydrolysis, and it is considered a promising natural compound for breast cancer prevention and treatment [29].

### 2.2. Lipids

Fatty acids play important roles in both biological and nutritional contexts. Fatty acids have received increasing attention from a clinical perspective for their involvement in the onset and progression of diseases, including cancer. Dietary lipids have already been proven to assume a pivotal role in the etiology of cancer, and different kinds of lipids can have other effects on cancer regulation [30]. Fatty acids are a type of lipid typically derived from fats and oils in natural sources such as triacylglycerols or phospholipids. They can be either saturated (SFAs) or unsaturated (UFAs) [31]. They have received increasing attention because of their involvement in the generation and progression of tumors [32].

#### 2.2.1. Saturated Fatty Acids

There is some evidence to suggest that lauric acid, a saturated medium-chain fatty acid from coconut oil, may exhibit antitumor activity mediated through oxidative stress-induced apoptosis [33]. Lappano et al. [34] demonstrated that lauric acid could promote the production of reactive oxygen species, activate the transduction pathways, and change genetic expression. Some evidence suggests that lauric acid may have antiproliferative and proapoptotic abilities in breast cancer and endometrial cancer cells. Thus, lauric acid has been related to some health-promoting advantages of coconut oil intake, such as improved life satisfaction in breast cancer patients during sickness [35].

#### 2.2.2. Monounsaturated Fatty Acids

Oleic acid is a common fatty acid found in the biosphere. It is a monounsaturated fatty acid that has 18 carbon atoms in its fatty acid chain. It is found in a variety of foods, including olive oil, avocado oil, and nuts. Furthermore, there is some evidence to suggest that oleic acid may inhibit the overexpression of HER2, a well-defined oncogene that plays a decisive part in the etiology, invasiveness, progression, and metastasis of human cancers [36]. Notably, the effects of oleic acid on cell growth and proliferation may vary depending on the type of cell and the context. While some studies have suggested that oleic acid may have antiproliferative effects on certain tumor cell lines, other studies have reported conflicting results [36]. Some studies have suggested that both oleic acid and α-linolenic acid may have inhibitory effects on colorectal cancer cells. The Mediterranean diet [37], which is characterized by high consumption of olive oil, has been connected with lower morbidity of multiple kinds of cancer [38]. Oleic acid is a main ingredient of olive oil, and it has been suggested that its chemopreventive properties may contribute to the protective effects of the Mediterranean diet.

#### 2.2.3. Polyunsaturated Fatty Acids

Naturally occurring polyunsaturated fatty acids (PUFAs) cannot be synthesized by the body and are only obtained from dietary sources. For this reason, PUFAs are also often referred to as “essential fatty acids”. PUFAs are classified into two main categories determined by the position of the first double bond from the methyl end of the carbon chain: omega-6 PUFAs and omega-3 PUFAs [39]. Omega-3 PUFAs, also known as O3FAs, are found primarily in natural ocean sources such as fatty fish, algae, and krill. The two most well-known omega-3 PUFAs are eicosapentaenoic acid (EPA) and docosahexaenoic acid (DHA).

O3FAs have well-established anti-inflammatory properties, which are relevant to cancer prevention and treatment. Additionally, O3FAs have been shown to have a variety of anticancer effects through different mechanisms [40], such as binding to specific proteins and altering the phospholipid fatty acid composition of cell membranes. PUFA supplements, particularly those containing EPA and DHA, are often used by cancer patients as a complementary or alternative therapy. Studies have shown that O3FAs, particularly EPA and DHA, have anticancer effects against colorectal cancer. A study by Volpato et al. [41] reported that a better intake of EPA and DHA was related to a lower rate of developing colorectal cancer. This is believed to be due to the ability of EPA and DHA to restrict the growth and proliferation of colorectal cancer cells. In addition, the anti-inflammatory and anticancer properties of O3FAs suggest that they may have potential as an adjunct therapy in combination with traditional cancer treatments such as chemotherapy and radiation [41].

### 2.3. Polysaccharides

Polysaccharides are one of the four basic substances for life, diffusely distributed in microbes, plants, and animals. Medicinal polysaccharides derived from plants have been investigated for their potential as adjuvant therapies in cancer treatment. Preclinical and clinical studies have shown promising results. These polysaccharides are often combined with radiotherapy and chemotherapy to enhance their efficacy and reduce toxicity. Plant polysaccharides with noticeable antitumor effects have been shown to come into play in tumorigenesis in different cell lines primarily by restraining tumor growth, inducing apoptosis, increasing immunity, and cooperating with iatrochemistry drugs [42,43] (Figure 4b,c).

Polysaccharides has an innate antitumor effect on tumor cells. The polysaccharide from *Laminaria japonica Aresch* could significantly inhibit the multiplication of nasopharyngeal carcinoma cells, and the suppression rate was enhanced with increasing polysaccharide concentration [44]. Apoptosis is a type of gene-mediated programmed apoptosis and a critical phenomenon that drugs may induce in anticancer therapy. The five new polysaccharides SLNT-5 were extracted from the fruiting body of Lentinus edodes. SLNT1 and JLNT1 could improve the suppression rates of H22-bearing mice by increasing serum IL-2 and TNF-α production and inducing cancer cell death [45]. Tumor immunotherapy is also classified as a tumor therapy strategy [46]. Some polysaccharides do not directly inhibit tumor cells but exhibit anticancer activity by increasing the body's immune functions. The immunomodulatory ability of polysaccharides is classified as the foremost mechanism of the anticancer effect. Xue et al. [47] proved that fucoidan can inhibit mammary carcinogenesis in rats through the PD1/PDL-1 signaling pathway. Polysaccharides are immune enhancers that activate the activity of immune cells, such as T cells, B cells, MT cells, NK cells, CTL cells, and LAK cells [48] (Figure 4d). Polysaccharides can stimulate macrophages to release TNF-α and NO. Furthermore, they also have antitumor effects and immunomodulatory effects on tumor-bearing hosts. Bamodu et al. [49] demonstrated that astragalus polysaccharides could enhance the M1 polarization of macrophages, the function of dendritic cells, and T-cell-mediated antitumor immune reactions in patients with lung cancer [49]. Therefore, the antitumor mechanisms of polysaccharides are not self-governed but are correlated with each other.

### 2.4. Vitamins

Nutritional supplements are considered to be a needed element for people with an unbalanced daily diet. Vitamins are organic compounds that are essential for maintaining health, and their corresponding supplements are the most commonly used dietary supplements, capable of promoting health and/or preventing and mitigating disease. In addition, vitamin A was the most popular complementary and alternative medicine preparation used [50], and vitamin supplementation may provide a relatively effortless method for tumor prevention in human populations. Vitamins A, C, and E have been most associated with cancer [51].

Vitamin A is the nutritional term for a kind of lipid-soluble unsaturated hydrocarbon, which can be obtained from the daily diet either as preformed vitamin A (primarily retinyl esters, retinol, and a minor amount of retinoic acid) or in the form of provitamin A carotenoids. Vitamin A and its derivatives mainly affect the differentiation of epithelia and other tissues and inhibit the proliferation of preneoplastic and neoplastic cells [52]. Both innate and adaptive immunity require vitamin A [53]. It not only influences the activation of neutrophils and macrophages but also manages the differentiation of T-helper cells and B cells [54], which may be used to slow down the carcinogenic process. In a promising analysis with 20 years of follow-up, Li et al. [55] found that women who consumed a high intake of carotenoids had a lower chance of developing breast cancer, meaning that carotenoids proved a more significant inverse association with the risk of breast cancer. Antioxidants such as vitamin A and carotenoids have cytotoxic effects on tumor cells and do not affect normal cells, thus minimizing the side effects of chemotherapy drugs.

Vitamin C, alternately named ascorbic acid or ascorbate, is a necessary water-soluble vitamin that plays an essential role in the life activities of the human body. It cannot be synthesized by the human body and therefore needs to be ingested as food [56]. Cancer can induce oxidative stress and ROS formation. Vitamin C is a major antioxidant, and the generation of free radicals from advanced cancer likely results in increased consumption of vitamin C, which results in dramatically lower plasma vitamin C levels in cancer patients than in ordinary people [57]. Restoring vitamin C levels to reasonable ranges in cancer patients may be a simple treatment method. Furthermore, vitamin C also has pro-oxidative effects that may cause damage to ROS, protein glycation, and DNA. At high concentrations, vitamin C exhibits the killing of multifarious types of tumor cells in in vitro and in vivo experiments via a noteworthy generation of ROS [58]. Schoenfeld et al. [59] testified that ascorbate could selectively sensitize non-small-cell lung cancer and glioblastoma cells, and preclinical studies and clinical tests also proved the possible efficacy of pharmacological ascorbate in these two species of tumor treatment.

Vitamin E, a lipid-soluble vitamin, was found in wheat germ oil in 1922, comprising eight natural isoforms, i.e., four isoforms of tocopherol and four isoforms of tocotrienol [60]. In all the inartificial isomeric forms of vitamin E, alpha-tocopherol is the predominant form in plasma and tissues, which is generally considered “the VE” in nutrition. Moreover, they must be absorbed from food sources, and tocopherols are abundant in vegetable oils, such as oils from soybeans, corn, sesame, cottonseeds, and nuts. Research has shown that low vitamin E levels are related to an increased risk of tumors [61]. The antitumor effects of tocopherols have been mainly ascribed to their antioxidant, anti-inflammatory, antiproliferative, antiangiogenic, and immune-modulatory mechanisms. Ju et al. [62] showed that dietary γ-TmT dramatically reduced colon carcinogenesis in AOM/DSS-group mice. They further suggest that c-TmT inhibited the increase in oxidative stress during colon tumorigenesis. Tocopherols could play an antiproliferative role by inducing cell apoptosis.

### 2.5. Minerals

A mineral is an essential micronutrient for the human body, which not only forms human tissues but also sustains fundamental physiological functions of the human body [63]. Numerous population studies have shown that dietary intake of different minerals could interfere with the development of various cancers [64]. Cancer may impede the regular intake of micronutrients. Additionally, the inflammatory activity of cancer and the catabolic effects of gastrointestinal symptoms or antitumor therapy may lead to malnutrition, reducing micronutrient intake [65]. The American Cancer Society Guide for Informed Choices states that taking standard mineral supplements during and after cancer treatment may have health benefits for patients [14].

Calcium is essential for healthy bones and teeth and for all living cells to maintain structure and function. Calcium signaling is also critical for cell cycle regulation in cancer cells because it participates in the regulation of cell growth, differentiation, and apoptosis [66]. The function of calcium is to regulate cell death pathways and restrain cell proliferation in breast cancer cell lines [67]. Calcium is necessary for the optimum activity of vitamin D, and there is evidence that the anticancer effects of calcium are partly mediated by vitamin D. Mathiasen et al. [68] showed that calcium is a crucial mediator of vitamin D compound-induced apoptosis-like death of breast cancer cells. Furthermore, vitamin D and calcium have a chemopreventive effect against breast cancer [69]. The prospective observational study from Keum et al. [70] showed that calcium supplements could serve as additional targets for colorectal cancer prevention.

Selenium is an essential trace element with remarkable chemical properties, including antioxidative, antimutagenic, antiviral, and anticarcinogenic properties. In 1957, selenium was recognized as an essential element for human health [71]. By 1969, Shamberger and Frost demonstrated that selenium was not a carcinogen but a cancer-preventing agent [72]. Selenium consumption at a dose of more than 64.4 µg/L has a positive effect on breast cancer survival among female patients undergoing surgery [73]. Higher levels of serum selenium are observably associated with a lower probability of colon cancer in women, indicating that selenium intake is a crucial factor in influencing cancer risk in a population of marginally low-selenium individuals [74]. The implicit role of selenium compounds in cancer therapy is centered around their ability to increase cellular oxidative stress. Research has shown that normal cells can cope with the increase in oxidant fluxes, while cancer cells have reached the limits of their ability to control oxidative stress. Taking advantage of these differences, generating more ROS through selenium compounds could provide a therapeutic advantage [75].

Iron is a necessary nutrient that plays a crucial part in hemoglobin synthesis, DNA synthesis, and energy metabolism in all mammals. In addition, it is an indispensable trace element for life support. Recent studies have also shown that cancer patients commonly suffer from anemia, regardless of whether they receive any treatment [76]. Many studies have shown that abnormal iron homeostasis is a marker of cancer [77]. Ferumoxytol, also known as Feraheme, is an FDA-approved nanometer material for iron deficiency treatment. Ferumoxytol treatment significantly reduced disease burden in a mouse leukemia model and in patient-derived xenografts bearing leukemia cells with low ferroportin expression [78].

### 2.6. Polyphenols

Polyphenols are a varied group of naturally occurring compounds found in varieties of plant-based foods, such as fruits, vegetables, whole grains, tea, coffee, and cocoa. There are over 8000 known polyphenols and their structure can vary widely, although they are typically categorized according to the amount of phenol rings they hold and their structural elements [79]. Numerous prospective studies have shown that polyphenol compounds can have significant effects on human health, including beneficial effects on various health conditions, such as cancer, neurodegenerative-related diseases, and cardiovascular system injury. Numerous in vitro/vivo experiments have demonstrated that polyphenols from diverse dietary sources could have a pivotal effect in delaying the occurrence and progression of cancer, decreasing cell proliferation, inactivating carcinogens, inducing cell apoptosis, and regulating immune function (Table 1) [80].

Green tea is the second most widespread drink expended in the world after water. It has been studied for its potential health benefits, particularly in reducing the risk of cancer. Green tea is abundant in polyphenolic compounds known as catechins, specifically epigallocatechin-3-gallate (EGCG). EGCG has been shown to have a range of potential anticancer effects in several forms of cancer, including breast cancer, lung cancer, prostate cancer, and colon cancer [93,94]. In addition, EGCG has been shown to have anti-inflammatory activities by suppressing the generation of inflammatory cytokines, and it can also act as an antioxidant by scavenging free radicals and reducing oxidative stress [95]. The structure of EGCG determines its strong antioxidant ability, and it may slow the spread of cancer [96]. Lee et al. demonstrated that EGCG can modulate the antioxidant pathways to enable the selective death of cancer cells [97,98]. In addition, expression of PD-L1 in cancer cells has a significant effect on tumor immune escape and cancer progression. Rawangkan et al. [99] indicated that EGCG can inhibit both IFN-γ– and EGF-induced PD-L1 expression through regulating two signaling pathways, JAK2/STAT1 and EGFR/Akt, in A549 and H1299 cells. Therefore, EGCG has been shown to target various components of the tumor microenvironment, including cancer stem cells, cancer-associated fibroblasts, and immune cells, to exert its antitumor effects.

### 2.7. Recommended Daily Diet

Interest in the potential of daily dietary guidelines in helping to lower the risk for diverse kinds of cancer dates back several decades [100]. Good nutrition can play a crucial role in maintaining health. Therefore, the authors have summarized a recommended daily intake of nutrient elements that can provide dietary recommendations for people (Table 2).

## 3. Nutrients-Based Materials from Food as Delivery Systems for Cancer Intervention

The effectiveness of nutrients in the prevention of cancer is dependent on the maintenance of the bioavailability of the active ingredients. After oral administration, just a fraction of the molecule remains effective. Due to insufficient gastric residence time, poor intestinal permeability and/or solubility, and unstable food handling or gastrointestinal conditions, these factors limit the nutraceutical molecules' activity and potential health benefits.

### 3.1. Protein Nanoparticles

**Figure 5:**
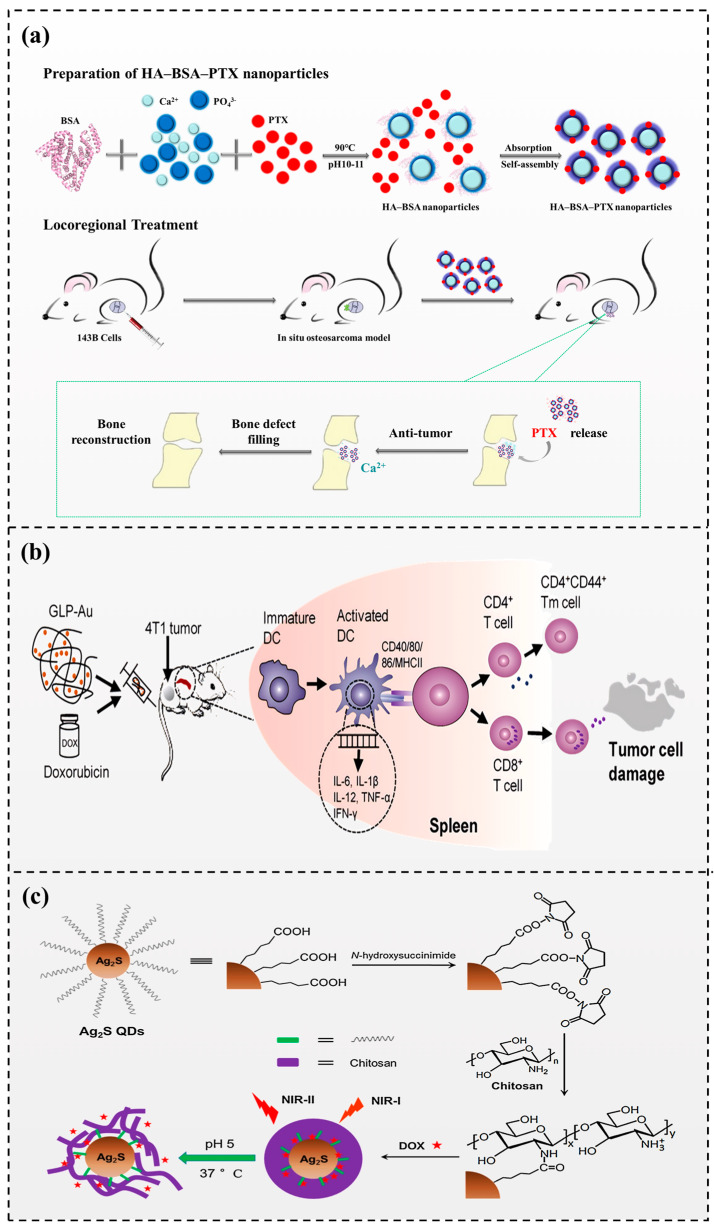
Nutrient-based delivery systems for cancer therapy. (**a**) The preparation procedure of the HA−BSA−PTX nanoparticles for enhanced tumor therapy [112] (reproduced with permission from publisher John Wiley and Sons). (**b**) Immunotherapy effect of polysaccharide gold nanocomposites in cancer [115] (reproduced with permission from publisher Elsevier). (**c**) The preparation procedure of the Ag_2_S(DOX)@CS nanospheres and mechanisms of pH-triggered DOX release and NIR imaging [116] (reproduced with permission from publisher Elsevier).

Biopolymers, especially dietary proteins, are commonly found in formulated foods due to their great nutritive value and general safety. Bovine serum albumin (BSA) is a spheroidal protein made of 583 amino acid residues with a molecular weight of probably 66,000 Daltons. An innovative in vitro study by Laursen et al. [110] indicated that BSA may suppress the proliferation of human breast cancer cells (MCF-7) by modulating the activities of autocrine growth regulatory factors. One of the critical properties of BSA is its ability to bind to a wide variety of small molecules, including drugs, fatty acids, and other ligands. Self-assembling nanoparticles composed of bovine serum albumin and paclitaxel have been developed and investigated as a possible drug delivery system for cancer cures. The BSA component of the nanoparticles provides a hydrophilic surface that can enhance drug solubility and prolong drug circulation time in the bloodstream. The resulting nanoparticles have been shown to have a particle size of 50–150 nm, which is within the range considered optimal for efficient tumor targeting [111]. Thus, Liu et al. [112] synthesized a novel ternary antitumor drug complex composed of hydroxyapatite (HA), BSA, and paclitaxel for the in situ therapy of osteosarcoma following surgery. The diameter of the drug complex nanoparticles was about 55 nm, and the drug delivery rate was 32.17 wt%. The nanoparticles also displayed the slow-release characteristics of paclitaxel and calcium ions and showed high biocompatibility with human fetal osteoblasts (hFOB1.19). In situ, osteosarcoma model research has shown that drug complex nanoparticles can exhibit remarkable antitumor effects and inhibit cancer metastasis (Figure 5a). Sun et al. [113] demonstrated that peptide/protein nanoparticles had great affinity. Fluorinated EGCG and Melittin were used to construct a composite nanoparticle through self-assembly, which had a synergistic antitumor effect. In addition, the nanoparticle can regulate PD-L1 and apoptosis signaling, thereby inhibiting tumor growth.

### 3.2. Polysaccharide Nanoparticles

The structures of polysaccharides could offer versatility in synthesizing multifunctional nanocomposites, and the polysaccharides extracted from natural herbs could be embedded into nanoparticles with immunoregulatory characteristics for increased efficacy in tumor treatment [114]. The nanocomposites were synthesized with Ganoderma lucidum polysaccharide and gold nanoparticles, which could more efficiently induce dendritic cell activation and robust T-cell responses than free polysaccharides. Nanocomposites also showed practical inhibitory effects on breast cancer tumor growth and lung metastasis when synergistic with doxorubicin [115] (Figure 5b). Chitosan has been widely used in various biomedical applications as a cationic polysaccharide. Tan et al. [116] designed a core-shell nanosphere, Ag_2_S(DOX)@CS, which contains chitosan (CS)-encapsulating silver sulfide quantum dots (Ag_2_S QDs) with entrapped doxorubicin (DOX). The nanospheres could be imaged and tracked for drug delivery, release DOX at a given pH in cancer cells, and have great anticancer ability. Fucoxanthin, a carotenoid-derived artificial compound conjugated with chitosan and glycolipid nanogels, significantly increased the cellular uptake and anticancer efficacy of fucoxanthin in human colon cells [117] (Figure 5c).

### 3.3. Solid Lipid Nanoparticles

Liposomes and polymeric nanoparticles are two of the most extensively studied drug delivery systems for cancer treatment, and they offer several advantages over traditional drug delivery approaches [118]. Effective cancer therapy requires selective drug delivery systems to overcome the limitations of traditional treatments. Moreover, they have been shown to be advantageous in anticancer drug delivery. Liposomes can increase the bioavailability of drugs and provide controlled release of the drug, enhancing its therapeutic effects and reducing its toxicity. With outstanding biocompatibility, inherent cancer-targeting abilities, and potential pharmacological properties, liposomes and polymeric nanoparticles are perfect candidates for designing high-performance drug carriers for cancer therapy.

UFAs have a connatural tumor-targeting ability and can increase the tumor accumulation of chemotherapy drugs through a prodrug strategy. In addition, the therapeutic action of paclitaxel in brain cancers is relatively limited by the blood–brain barrier. Incorporating UFAs into paclitaxel delivery systems could enhance the therapeutic potential of paclitaxel for brain cancer therapy. UFAs can enhance the accumulation of drugs in the brain due to their ability to pass through the blood–brain barrier [119]. The attachment of alkyl chains, such as UFAs, to medicinal molecules can increase their lipophilicity, which can help them penetrate the blood–brain barrier by promoting passive diffusion [120].

**Figure 6:**
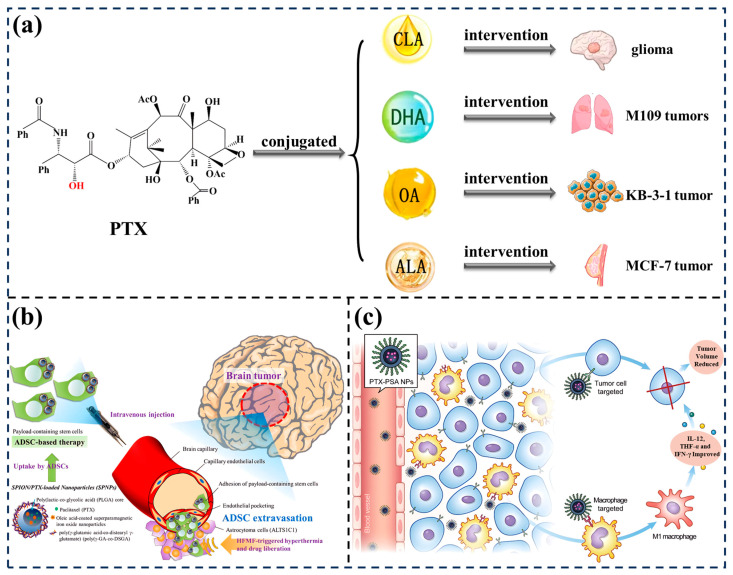
Synergistic anticancer activity of fatty acids with paclitaxel and potential activity of transporting chemotherapy drugs. (**a**) The therapeutic efficacy of conjugated Paclitaxel-CLA/DHA/OA/ALA [121,122,123,124]. (**b**) Schematic description of the ADSC-mediated delivery of SPNPs toward brain tumors for dual-modality treatment of orthotopic astrocytoma [125] (reproduced with permission from publisher Elsevier). (**c**) Schematic description of the PA-modified HSA paclitaxel nanoparticles targeting tumor cells and macrophages against breast cancer [126] (reproduced with permission from publisher Elsevier).

As shown in Figure 6a, conjugated linoleic acid was covalently related to paclitaxel. Compared with free paclitaxel, the cytotoxicity of the conjugate was lower, and the cellular uptake efficiency on glioma cells (C6) was higher. Additionally, unlike free paclitaxel, the conjugate may spread in brain tissue and retain higher accumulation levels throughout a period of 10 days. The anticancer ability of tumor-bearing rats after being administered the compound was dramatically higher than that of those treated with paclitaxel only [121]. Bradley et al. [122] indicated that covalent conjugation of paclitaxel with DHC could prominently change the pharmacokinetics and elimination of the drug. Compared with free paclitaxel, conjugated paclitaxel exhibited lower cytotoxicity and more significant antitumor activity in vivo. Luo et al. [123] indicated that the drug delivery system PEGylated-paclitaxel-oleic acid has a high drug-loading capability and can selectively release the drug at the tumor site. Xu et al. [124] indicated that α-linolenic acid paclitaxel conjugate nanoparticles enhance antitumor ability and have greater biocompatibility than small molecules alone. Therefore, benefiting from outstanding biocompatibility, inherent tumor-targeting abilities, and potential pharmacological action functions in cancer treatment, UFA-modified drug delivery strategies have been diffusely studied for the high-performance delivery of chemotherapeutics [125,126] (Figure 6b,c).

## 4. Food Nutrient Derivatives for Cancer Intervention

Chemical modification is a common means that is involved in the structural regulation of polysaccharides by chemical methods to gain polysaccharide derivatives with more or novel bioactivity. Chemical modification of polysaccharides could change their bioactivities by adding substituent groups, thereby enhancing their inherent biological activities as well as generating new biological functions [127].

**Figure 7:**
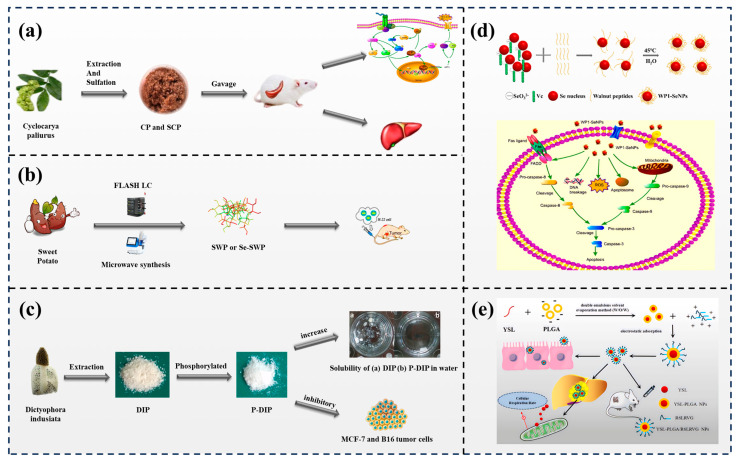
The derivatization of food nutrients. (**a**) Sulfated modification increases the immunomodulatory effect of cyclocarya paliurus polysaccharide [128] (reproduced with permission from publisher Elsevier). (**b**) Phosphorylated modification enhances the water solubility and anti−tumor activities of dictyophora indusiate polysaccharide [131] (reproduced with permission from publisher American Chemical Society). (**c**) Anti-proliferative activity of selenium−containing polysaccharides against tumor cells [135] (reproduced with permission from publisher Elsevier). (**d**) The preparation of WP1−SeNPs and possible signaling pathway to induce apoptosis in McF-7 cells [148] (reproduced with permission from publisher Dove Medical Press). (**e**)The chemical structure of the peptide, the preparation of YSL−PLGA/R6LRVG NPs, and the anticancer mechanism [150] (reproduced with permission from publisher Dove Medical Press).

Sulfation of polysaccharides is one of the most effective methods to change the anticancer ability of natural polysaccharides [128] (Figure 7a). Wei et al. [129] extracted a polysaccharide from *Radix edysari* and synthesized its sulfated derivatives. Compared with polysaccharides alone, all derivatives showed visible antitumor ability on A549 cells and gastric cancer cells (BGC-823). Sulfated modification can increase the antitumor ability of polysaccharides.

Selenium (Se) is a necessary trace element that is important for developing and maintaining physical health. Furthermore, Se can only be absorbed from food or other sources of supply [130]. Some studies have shown that selenium can dramatically increase immune-enhancing activity [131] (Figure 7b). Feng et al. [132] demonstrated that selenizing *Chuanminshen violaceum* polysaccharides dramatically enhanced the propagation of lymphocytes and promoted the generation of IFN and IL-4. Accordingly, selenylation modification of *Chuanminshen violaceum* polysaccharides could facilitate immune-enhancing activity. Wang et al. [133] reported that the selenized *Artemisia sphaerocephala* polysaccharide can observably raise the antitumor abilities of polysaccharide derivatives in vitro, thus representing powerful evidence for the application of polysaccharides.

Several investigations have indicated that the charged phosphate groups manufactured by phosphorylation not only increase the solubility and influence the molecular weight and chain structure of polysaccharides but also attach to receptors on the surface of macrophages with close affinity, boosting the immune system efficaciously and thus generating antitumor activity [134,135] (Figure 7c). Qian et al. [136] prepared a novel derivative of phosphorylated corn straw xylan, and compared with xylan alone, the phosphorylated structure had better thermal stability and crystallinity. In addition, the antioxidant ability and anticancer activity of the phosphorylated structure were more remarkable.

The stability of polyphenols relies on their management of modification and reaction [137]. Chen et al. [138] assembled metal–phenolic network nanoparticles containing poly (ethylene glycol) (PEG), ZrIV, and EGCG to deliver functional small molecules, which have been proven to achieve higher-capacity loading of anticancer drugs. Zhang et al. [139] proposed a FeOOH-assisted preparation strategy to construct metal–phenolic networks, which include FeCl_3_·6H_2_O and polyphenols. In addition, the FeOOH@Fe-polyphenol NPs can overcome the shortage of free polyphenols. When triggered by the tumor microenvironment, metal ions and polyphenols can be released in response, demonstrating their ability in the area of anticancer treatment. Wang et al. [140] constructed a multifunctional nanoparticle, Au@resveratrol, through the galvanic replacement reaction with HAuCl4 and resveratrol, which was confirmed to inhibit A375 cell division. Zhang et al. [141] constructed a drug delivery system of fluorinated EGCG, which can load small interfering RNA anti-TOX. Fluorinated EGCG can reduce the expression of PD-L1, resulting in excellent suppression of cancer growth as well as antimetastatic effects. Studies have demonstrated that EGCG can work synergistically with other natural functional components, such as curcumin or resveratrol, to enhance their anticancer effects. Additionally, EGCG has also been tested in combination with commonly used chemotherapeutic drugs, such as cisplatin and doxorubicin, to improve treatment outcomes and reduce side effects (Table 3). Furthermore, nanoparticles have been used as a drug delivery system for EGCG to enhance its bioavailability and target specific cancer cells. The carboxymethyl chitosan-grafted EGCG with AuNP nanocomposites developed by Yuan et al. [142] could potentially enhance the anticancer action of EGCG in vivo. EGCG-conjugated poly(ethylene glycol) and chlorin e6 of polyphenol nanoparticles were developed as a delivery system for EGCG in photodynamic cancer therapy, and it was found to have improved antitumor efficacy [143]. In summary, reasonable modification of polyphenols could significantly increase their anticancer ability.

Peptides used for therapeutic needs have several advantages over larger biomolecules. One of the major advantages is their ability to be easily synthesized and chemically modified. This allows for the development of a wide range of peptide-based drugs with different structures and functions [25]. Liao et al. [148] designed a simple and convenient method to obtain walnut peptide-functionalized SeNPs by anchoring peptides on the surface of the SeNPs. Moreover, the antiproliferative ability of the composite structure was dramatically increased compared with that of free peptides and SeNPs. The composite structure shows selectivity between tumor and normal cells, with apoptosis-inducing activities on MCF-7 cells (Figure 7d). Keykanlu et al. [149] synthesized perfluorooctyl bromide (PFOB) nanoparticles for bee venom melittin and lactoferrin delivery in camel milk. The better therapeutic effect of nanoparticles was demonstrated by in vitro experiments, which increased MCF-7 cell death. Tyroserleutide is extracted from the pig spleen, which could observably extend the lifespan of mice implanted with mouse hepatoma cells (H22). An in vitro study demonstrated that the R6LRVG-functionalized tyroserleutide-PLGA nanoparticles had enhanced cellular uptake compared to nonfunctionalized nanoparticles. Furthermore, the functionalized nanoparticles showed improved permeability across an intestinal barrier model, suggesting their potential for oral drug delivery. The compound can provide a helpful oral delivery system for tyroserleutide and might represent a novel strategy for the oral delivery of food-derived bioactive peptides for cancer interventions [150] (Figure 7e). These results suggest that food protein-derived peptides may be promising for food and pharmaceutical applications.

## 5. Conclusions and Challenges

A healthy diet can play a significant role in decreasing the incidence of chronic diseases such as cancer. Dietary interventions can enhance the efficacy of chemotherapy and reduce the toxicity of chemotherapy drugs. It is also a crucial assistive method for cancer therapies. There is a large body of evidence supporting the potential of dietary modification and nutrient supplementation for cancer prevention and intervention. Many prospective cohort studies have shown that certain foods and nutrients (protein, fatty acids, polyphenols, vitamins, minerals, fruits and vegetables, fish, white meat, and whole grains) can help reduce the risk of cancer. Overall, nutritional interventions can be an effective and relatively low-cost approach to improving patient outcomes and satisfaction with body weight changes and quality of life. However, it must be noted that nutritional interventions should be tailored to each patient's individual needs and preferences and should be implemented in conjunction with other therapies and lifestyle changes as needed.

Primarily, it is crucial to correctly identify the subject of nutritional interventions in cancer research. The populations in low-income nations, constrained by the level of socio-economic development, find it challenging to ensure access to a wide variety of foods and nutrients, notably fresh fruits and vegetables. The ubiquitous condition of nutritional deficiency, or even its stark absence, necessitates targeted nutritional supplementation interventions, thus rendering them appropriate and rational. Although residents of high-income nations generally have a sufficient baseline nutritional supply, there exist segments suffering from specific nutrient deficiencies. Secondarily, it is paramount that dietary and nutritional interventions commence at the earliest opportunity and persist throughout the life course with unwavering dedication for the outcomes to materialize. Lastly, it has been illustrated that combined therapy for cancer is safer and more efficacious compared to monotherapy. This concept is no exception when it comes to the nutritional chemoprevention of cancer and anti-cancer foods. Bearing in mind that nutritional supplements and anti-cancer foods are often consumed over an extended period by asymptomatic healthy individuals or those at high risk, these formulations must demonstrate minimal toxic side effects or lack any severe toxic ramifications. Cancer represents a category of profoundly intricate diseases involving multifactorial influences, the participation of multiple genes, the emergence of numerous lesions, and evolutionary stages, often necessitating a prolonged development process spanning several years or even decades. It is difficult to envisage that short-term supplementation of individual nutrients or anti-cancer foods could restrain, decelerate, or reverse the carcinogenic effects. Furthermore, combined usage and formulations can reduce toxic side effects, hence making compound usage undoubtedly desirable.

In summation, cancer is a manifestation of the combined effects of factors such as biological inheritance and healthcare services. The dietary patterns and nutritional status of the population play a central role in the incidence and progression of cancer. Prevention or postponement of the onset and progression of cancer can be achieved by adopting a balanced diet, ensuring nutritional equilibrium, engaging in active physical activities, and maintaining a healthy weight.

## Figures and Tables

**Table 1:** Classification of dietary polyphenols and anticancer mechanisms.

	Category		Experimental Model	Molecular Mechanism	Ref.
	Scaffold	Compound			
Flavonoids	Flavone 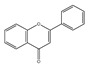	Apigenin (celery) 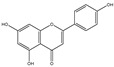	Mice model of pancreatic cancer.	Apigenin induces SHIP-1 expression, reduces inflammatory tumor-derived factors, and enhances anti-tumor immune responses.	[81]
	Flavonol 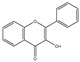	Quercetin (onion) 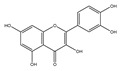	Adrenocortical carcinoma cell—H295R, SW-13. Mammalian epithelial nontumoral cells—MCF-10A	A higher cytotoxicity response on both cancerous cell lines.	[82]
	Flavanone 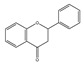	Naringin (pomelo) 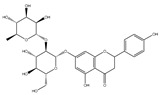	Mice model of glioblastoma cancer.	Naringin inhibited invasion and angiogenesis, making the tumor grow more slowly.	[83]
	Isoflavonoid 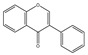	Genistein (soybean) 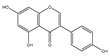	Human laryngeal cancer cells—TU212, Hep2.	Genistein suppresses laryngeal cancer cell survival through the p53-miR-1469-Mcl1 pathway.	[84]
	Flavanol 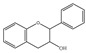	(+)-Catechin (green tea) 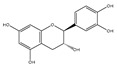	Non-small-cell lung cancer cell—A549.	Catechin inhibits the proliferation of A549 cells through regulating its cell cycle arrest or indirectly via the p21 signaling pathway.	[85]
	Anthocyanidin 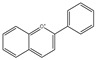	Cyanidin (purple sweet potato) 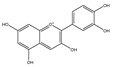	Human non-small-cell lung cancer—SPCA-1.	Cyanidin induces early apoptosis of NSCLC cells by regulating Caspase-3, Bax, Bcl-2, and P53.	[86]
Non-Flavonoids	Benzoic Acid Derivates 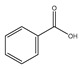	Protocatechuic acid (Illicium verum) 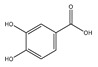	Human colorectal carcinoma—ATCC, CCL-247.	Protocatechuic acid inhibits the proliferation of the colon cancer cell line.	[87]
	Cinnamic Acid Derivates 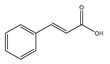	Caffeic Acid (coffee) 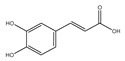	Mice model of osteosarcoma.	Caffeic acid can inhibit tumor mass formation.	[88]
	Stilbenes 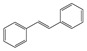	Resveratrol (peanut) 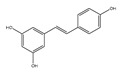	The LoVo cell line—colon adenocarcinoma cell line, Dukes’ type C, grade IV, ATCC^®^CCL-229™.	Resveratrol modulates SIRT gene expression and exert anticancer activity in colon cancer cells and cancer stem-like cells.	[89]
	Curcuminoids 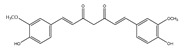	Curcumin (ginger) 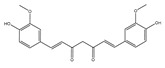	Mice model of cervical cancer.	Curcumin inhibits the growth of HeLa cell xenografts in mice through up/downregulating the expression of numerous proteins.	[90]
	Lignans 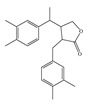	Pinoresinol (sesame) 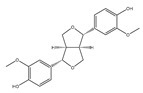	Human Colorectal cancer cells—RKO, SW480, and HCT116.	Pinoresinol induces cell cycle arrest and apoptosis in CRC cells by inducing the ATM–p53 axis.	[91]
	Tannins (grape) 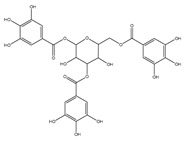		Human cervical carcinoma cells—HeLa (CCL-2).	Tannin inhibits the proliferation of HeLa cells by inducing cell cycle arrest in the G2/M phase along with ROS-mediated activation of the mitochondrial apoptotic pathway.	[92]

**Table 2:** The recommended daily intake of nutrient elements.

Nutrient Elements		Recommended Daily Dietary	Ref.
Protein		Adult: 0.8 g/(kg·d); Older persons: 1.2 g/(kg·d), Supplement: leucine.	[101]
Fat		30 to 35% of total energy intake, replacement of SFA with PUFA and MUFA, and avoidance of industrial TFA.	[102]
Carbohydrates		50% energy from carbohydrates being derived from food.	[103]
Vitamins	Vitamin A	Adult men: 800 μg/day; Adult women 700 μg/day. Take less than 3000 μg/day to avoid damaging the liver	[104]
	Vitamin C	Adult: 80 mg/day	[105]
	Vitamin E	Adult: 5–15 mg/day	[106]
Minerals	Calcium	Adult: 800 mg/day	[107]
	Selenium	Adult: 55–60 μg/day	[108]
	Iron	Adult: 16 mg/day	[109]

**Table 3:** The combined anticancer actions of EGCG.

Health-Related Properties		Experimental Model	Mechanism	Ref.
Acts synergistically with natural compounds	Curcumin	Mice model of colorectal carcinoma.	Curcumin in combination with EGCG attenuates the tumor CM-induced transition of NECs toward TECs by inhibiting the JAK/STAT3 signaling pathway. Furthermore, combining curcumin and EGCG reduces tumor growth and angiogenesis in the colorectal carcinoma PDX mouse model. The combined anti-angiogenic effect is better than that of curcumin or EGCG alone.	[144]
Combination with chemotherapeutic drugs	Doxorubicin	Mice model of osteosarcoma.	EGCG targeting LncRNA SOX2OT variant 7 produces synergistic effects with doxorubicin on osteosarcoma cell growth inhibition. On the one hand, EGCG could reduce doxorubicin-induced pro-survival autophagy by decreasing SOX2OT variant 7 to improve the growth inhibition of doxorubicin. On the other hand, EGCG could partially inactivate the Notch3/DLL3 signaling cascade targeting SOX2OT variant 7 to reduce the stemness and then abate the drug-resistance of osteosarcoma cells.	[145]
	Cisplatin	Bile duct carcinoma cell lines—CCSW1, BDC, EGI1, SkChA-, and TFK1. Gallbladder cancer cell lines—MzChA-, MzChA2, and GBC.	EGCG reduces the mRNA levels of various cell cycle-related genes, while increasing the expression of the cell cycle inhibitor p21 and the apoptosis-related death receptor 5. It also displays a synergistic cytotoxic effect with cisplatin in most tested BTC cell lines.	[146]
	Sunitinib	Mice model of breast cancer and lung cancer.	EGCG enhances the anti-proliferation and VEGF secretion-reducing effects of sunitinib in the three tested cell lines. EGCG treatment downregulated IRS-1 levels and suppressed mitogenic effects. EGCG potentially synergizes with sunitinib due to its ability to suppress the IRS/MAPK signaling induced by sunitinib.	[147]

## Data Availability

The original contributions presented in the study are included in the article, further inquiries can be directed to the corresponding author.

## Abbreviations

Akt	Protein kinase B
AOM/DSS	Azoxymethane/Dextran Sulphate Sodium
PGE2	prostaglandin E2
Bax	Bcl2 Associated X protein
Bcl-2	B-cell lymphoma 2
c-Jun JNK	c-Jun N-terminal kinase
COX-2	Cyclooxygenase-2
HepG-2	Hepatocellular carcinoma cell lines
HER2	Human epidermal growth factor receptor 2
IL-1β	Interleukin-1β
IL-2	Interleukin-2
IL-6	Interleukin-6
Mcl1	Induced myeloid leukemia cell differentiation protein
mTOR	Mammalian target of rapamycin protein
NF-κB	Nuclear Factor kappa B
NIH/3T3	3-methylcholanthrene-treated fibroblast cells
NO	Nitric Oxide
PARP	Poly [ADP-ribose] polymerase 1
PGE2	prostaglandin E2
PI3K	Phosphoinositide 3-kinase
ROS	Reactive oxygen species
SGC-7901	Gastric adenocarcinoma cells
STAT3	Signal transducer and activator of transcription 3
TNF-α	Tumor Necrosis Factor-α
uPA	Urokinase-type plasminogen activator
VEGF	Vascular endothelial growth factor
α-LA	α-Lactalbumin
γ-TmT	Gamma-tocopherol methyl-transferase
